# Perceived Support for Recovery and Level of Functioning Among People With Severe Mental Illness in Central and Eastern Europe: An Observational Study

**DOI:** 10.3389/fpsyt.2021.732111

**Published:** 2021-09-21

**Authors:** Catharina Roth, Michel Wensing, Jan Koetsenruijter, Ana Istvanovic, Antoni Novotni, Aleksandr Tomcuk, Jovo Dedovic, Tatijana Djurisic, Milos Milutinovic, Martina Rojnic Kuzman, Raluca Nica, Sarah Bjedov, Sara Medved, Tiberiu Rotaru, Bethany Hipple Walters, Ionela Petrea, Laura Shields-Zeeman

**Affiliations:** ^1^Department of General Practice and Health Services Research, Heidelberg University Hospital, Marsilius Arcades, Heidelberg, Germany; ^2^Croatian Institute of Public Health, Rockefellerova, Zagreb, Croatia; ^3^University St. Cyril and Methodius, University Clinic of Psychiatry, Skopje, North Macedonia; ^4^Health Institution Special Psychiatric Hospital Dobrota Kotor, Mental Health Promotion and International Cooperation Department and Department of Forensic Psychiatry, Kotor, Montenegro; ^5^Public Health Institute of Montenegro, Podgorica, Montenegro; ^6^Zagreb University Hospital Centre and the Zagreb School of Medicine, Zagreb, Croatia; ^7^Institute Liga Romana Pentru Sanatate Mintala, Bucuresti-Sector, Romania; ^8^Zagreb University Hospital Centre, Clinic for Psychiatry and Psychological Medicine, Zagreb, Croatia; ^9^Siret Psychiatric Hospital, Psychotherapy Unit, Siret, Romania; ^10^Trimbos Institute (Netherlands Institute of Mental Health and Addiction), Department of Mental Health Prevention and Expertise Centre for Tobacco Control, Utrecht, Netherlands; ^11^INSIGHT International Institute for Mental Health and Integrated Health Systems, Amsterdam, Netherlands

**Keywords:** mental health, severe mental illness, Eastern Europe, functional limitation, recovery

## Abstract

**Background:** Many people with severe mental illness experience limitations in personal and social functioning. Care delivered in a person's community that addresses needs and preferences and focuses on clinical and personal recovery can contribute to addressing the adverse impacts of severe mental illness. In Central and Eastern Europe, mental health care systems are transitioning from institutional-based care toward community-based care. The aim of this study is to document the level of functioning and perceived support for recovery in a large population of service users with severe mental illness in Central and Eastern Europe, and to explore associations between perceived support for recovery and the degree of functional limitations.

**Methods:** The implementation of community mental health teams was conducted in five mental health centers in five countries in Central and Eastern Europe. The present study is based on trial data at baseline among service users across the five centers. Baseline data included sociodemographic, the World Health Organization Disability Assessment Schedule (WHODAS 2.0) for functional limitations, and the Recovery Support (INSPIRE) tool for perceived staff support toward recovery. We hypothesized that service users reporting higher levels of perceived support for their recovery would indicate lower levels of functional limitation.

**Results:** Across all centers, the greatest functional limitations were related to participation in society (43.8%), followed by daily life activities (33.3%), and in education or work (35.6%). Service users (*N* = 931) indicated that they were satisfied overall with the support received from their mental health care provider for their social recovery (72.5%) and that they valued their relationship with their providers (80.3%). Service users who perceived the support they received from their provider as valuable (*b* = −0.10, *p* = 0.001) and who reported to have a meaningful relationship with them (*b* = −0.13, *p* = 0.003) had a lower degree of functional limitation.

**Conclusion:** As hypothesized, the higher the degree of perceived mental health support from providers, the lower the score in functional limitations. The introduction of the community-based care services that increase contact with service users and consider needs and which incorporate recovery-oriented principles, may improve clinical recovery and functional outcomes of service users with severe mental illness.

## Introduction

Severe mental illness, such as severe major depression, bipolar disorder, schizophrenia, schizophreniform, and schizoaffective disorder affect quality of life and functioning in all aspects of life: personal, professional, and social (1). People living with severe mental illness (SMI) are more likely to die prematurely, to experience somatic comorbidities, and to suffer from poor physical health compared to the general population (2). Additionally, they are at higher risk of having a low quality of life due to poverty, social exclusion, stigma and discrimination (3, 4). People with SMI often experience problems in multiple life domains, including personal and social functioning and persistent clinical symptoms, which may require frequent or lengthy admission to psychiatric inpatient treatment (5–7). Access to high-quality mental health care remain far from ideal across the world (8–11). To advance progress, in 2013 the World Health Organization (WHO) developed and introduced the Mental Health Action Plan to improve mental health care worldwide (12), advocating for deinstitutionalization and encouraging the development and advancement of community-based mental health services as a way to improve treatment and care outcomes and upholding the rights of service users. In Central and Eastern Europe, many mental health systems are currently transitioning from delivering primarily institutional-based care toward offering more community-based care options and a greater diversity of treatment and care options [for a more detailed description of the healthcare systems in the five implementation sites, see Table 2 in the study protocol (13) or in Supplementary Material 2]. Longer length of stay in psychiatric hospitals or inpatient care may disrupt social, professional and community activities and impact participation and integration (7, 14, 15). It is anticipated that this transition to greater availability of community-based alternatives may support personal, social and community functioning as well as clinical and personal recovery (16, 17).

The recovery model of mental healthcare posits that patients can recover from their mental illness (REF).This model has been described as an individual process, journey, or experience (18); this processes focuses on (re)gaining control over one's life, rather than complete elimination of mental health symptoms (19) and is guided and supported by professionals, often by a multidisciplinary team of mental health professionals such as social workers, psychologists, and psychiatrists. The recovery process has shown to be enhanced by strong relationships with care professionals, by meaningful employment, and by financial security (18, 20, 21). Recovery involves the development of a new meaning and purpose of life, while dealing with the effects of mental illness (22). While each patient develops their own goals for recovery, common recovery goals focus on clinical recovery (remission of mental illness), functional recovery (meaningful participation in society), and personal/social recovery (being able to pursue a career or to study, living independently, participate in meaningful social activities, and restoring ones' personal identity) (23, 24). The recovery process of patients with (severe) mental illness may include finding ways to meaningfully participate in the community through employment, volunteering, and through involvement in social events, working to overcoming stigma, assuming more personal or professional responsibilities, and/or independently carrying out daily activities (25). Recovery is an active process for both patients and their mental health professionals (11, 26).

Community-based mental healthcare services that focus on recovery can be beneficial for personal and social recovery (27, 28). Support perceived as helpful or valuable by the service user involves an open attitude regarding their individual values, needs and preferences. Recovery-oriented service is based on the person-centered care model (29, 30), where care is provided in a collaborative way, taking into account the experiences and views of people with mental illness, especially with regard to their preferences for treatment options which need to be respected and accepted (11). Professional knowledge and experience can be used to deal with daily challenges of life in general and the unpredictability of an individual's process of recovery (26). Providing recovery-oriented care implies a reorientation from a guiding and decisive role toward a more supportive role of helping people with mental illness gain control over their lives and overcome struggles related to their disease. The therapeutic relationship between mental health care provider and service user has a significant impact on the process of recovery and thus on the success of the support provided (11, 31, 32).

Increasingly, mental healthcare systems throughout Europe are working toward embedding care delivery paradigms and processes that are focused on realizing recovery goals and facilitating recovery journeys of people living with a SMI (33, 34). The actual implementation of recovery-oriented care approaches in practice, however, remains challenging (13, 33), particularly for mental health care systems in transition (23, 24, 35). Furthermore, when care and treatment is primarily focused on lengthy inpatient care (27, 36), it can lead to challenges in maintaining a sense of purpose, which can make formulation of recovery goals challenging (37) and can affect self-identity (37). Designing and implementing holistic mental health care and support implies not only adopting approaches that facilitate individual goal setting and planning of treatment, but also delivering care within the service user's personal and social context, where recovery goals related to restoration of social identity and roles can potentially be formulated and realized.

As outlined above, personal and social recovery (recovery of self-identity and restoration of a sense of purpose and meaning) can be supported through the delivery of community-based services that embed recovery-oriented principles into care (27, 28). The aim of this study was to document the level of functioning and existing perceived support for the recovery process at baseline among a large population of mental health service users with severe mental illness in Central and Eastern Europe, and to explore associations between perceived support for recovery and the degree of functional limitations. Our hypothesis was that mental health service users reporting qualitatively higher levels of perceived support for their recovery would indicate lower levels of functional limitations.

## Materials and Methods

This study is based on the RECOVER-E project “*Large-scale implementation of community-based mental health care for people with severe and enduring mental ill health in Europe*” (38). The project was initiated in 2018 and includes five randomized trials evaluating the (cost-) effectiveness and implementation of community-based mental healthcare for people with severe mental illness (SMI) across five sites in Eastern and Central Europe. Among service users, it involves measurements at three time points: baseline, 12-months follow up, and 18-months follow up (38). A detailed description of the RECOVER-E project can be found elsewhere (38).

### Care as Usual for People With SMI in the RECOVER-E Project Sites

Prior to the RECOVER-E project, psychiatric care in **Zagreb, Croatia** relied on the conventional in-hospital treatment model, partially embedding clinical recovery-oriented practices. More specifically, the development of the treatment plan for service users was usually done by the treating psychiatrists, mainly focused on medication. Service users were not asked about their preferences and shared decision making (39) was only partially implemented in clinical care. Care did not focus on strengths of the service user. In 2016, the Ministry of Health and the Croatian Institute for Public Health led the project “Ensuring optimal health care for people with mental illness” with the Trimbos Institute in the Netherlands aiming to improve the quality of mental healthcare (40). This project resulted in the organization of three community mental health teams in Croatia (2017–2019), attempting to introduce an adapted model of the flexible assertive community treatment model for severe mental illness (41).

In **Kotor, Montenegro**, standard mental healthcare for people with SMI has been dominated by short and long inpatient hospital admissions for acute episodes, without any significant outreach or community services. A prior European Commission project (Twinning Light Project) in partnership with the Ministry of Health of Montenegro and the Trimbos Institute was implemented in 2013–2014; the priejct resulted in the initiation of a pilot flexible Assertive Community Team (ACT) within the Specialized Psychiatric Hospital Kotor, which operated for about a year and half. This multidisciplinary team consisted of psychiatrists, mental health nurses, a psychologist and a social worker. There was no significant support from the hospital management and no recognition of its work as a part of a regular service offered, therefore team members had additional clinical duties outside the pilot ACT team, were not paid for their work on the team and had to do these activities in their spare time. The initiative gradually eroded over time and then stopped.

In **Siret** County, **Romania**, the RECOVER-E project marked the first time a community mental health team was introduced as a service delivery model. Mental health care was provided only through the psychiatric hospital as ambulatory services by a consulting psychiatrist. Prior to the project, treatment plans for service users were not implemented with the exception of a pharmacological treatment plan. Shared decision-making was not part of the standard care approach. There were only a few service users that discussed their recovery goals with their health care provider, and nurses do sometimes focus on service user strengths and goals, but these practices were not carried out consistently.

In **Skopje, North Macedonia**, multidisciplinary community mental health trams existed before the project started. These teams were mostly composed of a psychiatrist and a psychiatric nurse; at some institutes, a psychologist and a social worker were also part of the CMHTs. Peer workers were not included prior to the RECOVER-E project. The teams had a caseload of shared service user, but the psychiatrist was the overall decision-maker and held overall responsibilities. Regular team meetings were held. Home visits or care consultations in public places such as parks were newly implemented within the RECOVER-E project and had not been done before. In addition, medical treatment focused mainly on medications, some forms of psychotherapy (supportive therapy, family psychoeducation, individual, and group therapy), and occupational therapy. Treatment plans were only agreed on verbally, not on paper, which was done during RECOVER-E (42).

In **Sofia, Bulgaria**, treatment for people with SMI was mainly provided by outpatient departments of hospitals. Admission to inpatient care was the standard protocol during any deterioration in mental health status for service users with SMI. Outpatients were usually monitored by psychiatrists, who consult a psychologist if necessary. In 2018, 12 state psychiatric hospitals, 12 mental health centers, 16 psychiatric wards in general hospitals, and six University psychiatric clinics were available across Bulgaria. Some additional outpatient services are run by the State Psychiatric Hospitals, Mental Health Centers, and by University Psychiatric Clinics. Some non-governmental organization (e.g., Global Initiative on Psychiatry in Sofia) run other mental health facilities. These facilities provide mainly outpatient services (43) delivered within clinical settings, meaning that there are no outreach activities or home treatment options available.

### Study Design

The study design for this paper is a quantitative analysis of baseline trial data aggregated across the five sites prior to the implementation of the CMHTs. The trials were staggered over time to optimize use of resources and enable lessons learned in implementation process.

### Study Setting

The study was conducted within mental healthcare hospitals in the five sites, which were described above. The rationale and selection criteria for these sites is described in the study protocol (38).

### Participants

Mental health service users between 18 and 65 years old, diagnosed with either bipolar disorder, severe major depression, schizophrenia, schizophreniform, or schizoaffective disorder according to the International Statistical Classification of Diseases and Related Health Problems (ICD-10) were eligible to take part in this study. Service users were excluded if they were under the age of 18, if they did not give their consent or were not able to give consent to participate or had compulsory treatment orders administered, also known as mandated treatment.

### Sampling

The main study (38), was powered on 90 service user per study arm to detect a clinically relevant effect on the primary outcome (functional status, measured with WHODAS 2.0, see below), thus the sample size per project site is 180. Therefore, the analysis of the aggregated data was based on a total sample of 900 participants. Recruitment was originally planned based on a three-step recruitment process, which aimed at first admissions, then re-admissions, and finally service user with short treatment history. However, few service user could be recruited in this way. Therefore, all service user attending the hospital (e.g., for routine consultations) who met the inclusion criteria were included in the study.

### Data Collection

Baseline data was collected by the local research teams in each site using a survey between December 2018 and April 2020. The survey included sociodemographic questions, measures to assess perceived support for the recovery process, INSPIRE (33) and functioning, which was measured using the WHO Disability Assessment Schedule 2.0 (WHODAS 2.0) (44). Questionnaires with no validated version in the local language were translated into the local language by members of the local research team who were fluent in English and in the local language using forward/backwards translation (38). Completing the questionnaire took the participants ~30–60 min.

## Measurements

### Sociodemographic Data Questionnaire

Individual characteristics (year of birth, sex, length of time using mental health service, employment status, marital status, level of education, and monthly income) were collected by using conventional standardized questions. The main diagnosis was obtained from service user records in the hospitals.

### INSPIRE (Recovery Support)

INSPIRE was developed by King's College London due to the lack of standardized user-related surveys on staff recovery support (33, 45). In this study, the full version (27-items) was used. INSPIRE was developed with input from service users, mental health care providers and researchers based on the conceptual framework for personal recovery in mental health (25). The conceptual framework consists of the following five categories, Connectedness, Hope, Identity, Meaning and Purpose, and Empowerment (25). The INSPIRE measure consists of two sub-scales (Support Scale, Relationship Scale) and assess service user's experiences of support they receive from their mental health providers for their recovery (33). The Support sub-scale (20 items) indicates how a mental health care provider can support a service user's recovery based on what is important to them within five categories: (1) Connectedness, which includes statements like “*An important part of my recovery is feeling supported by other people*”; (2) Hope refers to one's belief in recovery, motivation to change or the importance of having dreams for the future (e.g. “*Feeling hopeful about my future*”). (3) Identity includes statements regarding individual beliefs (e.g., “*An important part of my recovery is feeling I can deal with stigma or feeling good about myself* ”); (4) Meaning in life, includes statements such as understanding one's own mental illness, rebuilding one's life after difficult experiences (e.g., “*Having a good quality of life*”). (5) Empowerment refers to statements like the feeling to have one's life under control, trying new things, and building on one's strengths (e.g., “*Being able to manage my mental health*”) (25). Responses were coded as yes (*important*) or no (*not important*). If yes (*important*) was selected, the amount of support received was rated on a 5-point Likert scale from 1 (*not at all*) to 5 (*very much*).

The Relationship sub-scale (7 items) measures quality of the relationship between provider and user which is the key for the support received to be of value for the user (33) including statements such as “I feel listened to by my worker” or “I feel supported by my worker.” Responses were coded based on a 5-point Likert scale from 1 (*strongly disagree*) to 5 (*strongly agree*). The scales rank from 0% (no *recovery support*) to 100% (*highest support for recovery*).

INSPIRE Support scores lower than 72% indicate that support might not be perceived as helpful by the service user and INSPIRE Relationship scores lower than 78% indicate that the quality of the relationship between service provider and user is perceived as insufficient and could be improved (33, 46). INSPIRE shows adequate psychometric properties (Support sub-scale: internal consistency range 0.82–0.85, Relationship sub-scale: internal consistency 0.89) (33).

### WHODAS 2.0 (Functional Limitations and Level of Disability)

The World Health Organization Disability Assessment Schedule (WHODAS) 2.0 is a generic measure designed to assess functioning, disability, and health-related quality of life developed by the WHO (44). It is based on the concepts of the International Classification of Functioning, Disability and Health (ICF) and consists of six domains: Cognition (understanding and communication), Mobility (moving and getting along), Self-care-hygiene (dressing, eating, and staying alone), Getting along (interacting with other people), Life activities (domestic responsibilities, leisure, work, and school), Participation in society (joining in community activities) (47). In this study the 36-item version was used. Answering categories are none (1), mild (2), moderate (3), severe (4), and extreme (5, 47). For each functioning domain a specific score is produced where higher scores indicate a higher degree of functional limitation (0 no disability; 100 full disability) (44, 47). The WHODAS 2.0 demonstrates excellent psychometric properties (Cronbach's alpha between 0.94 and 0.98) (48, 49) and was found to be highly reliable based on test–retest studies in countries across the world (47).

## Ethical Considerations and Informed Consent Procedure

A central study protocol and a Data, Safety and Management Plan was developed by the project team to coordinate all of the five trails. Based on the central study protocol each site developed a separate study protocol. Each study protocol comprised a dedicated section as to how to prevent the risk of enhancing vulnerability and stigmatization (38).

### Ethical Approval

Ethics approval was obtained of the Medical Ethics Committee of the Medical Faculty Heidelberg (S-496/2018) prior to the start of the study in August 2018 by the Heidelberg Team. Additionally, each study in each implementation site has received ethical approval from a local institutional review board prior to the start of the study. An informed consent was signed by each participant prior to start of the study.

### Informed Consent Procedure

In each of the five sites, service users who decided to participate in the study were informed about the nature and the aim of the study by the local research team. Information letters including contact details of the local principal investigator and written consent forms were handed out and explained to the participating service user prior to the start of the study. The informed consent was signed by each participant. The documents are stored at a secure place according to the locally adapted version of the centralized project Data, Safety, and Management Plan. A copy of the information letter and the signed consent form was given to the participants.

The study was performed in accordance with the ethical standards as laid down in the 1964 Declaration of Helsinki and its later amendments.

### Data Analysis

The baseline data of the study population included in this project was analyzed using a multilevel regression model. The primary outcome, WHODAS 2.0 (44, 48, 49), was regressed on the INSPIRE measure (33). The analysis was used to explore associations between perceived support for recovery and the degree of functional limitations adjusted for other factors. Prior to analysis, all variables were checked for data entry errors and missing values. Descriptive statistics were used to calculate the means and standard deviations for continuous variables and frequencies and percentages for categorical variables in the sociodemographic data. Missing data were relatively modest with a maximum of 10.4% on income and 4.8% on marital status. To handle these missing data, a multiple imputation procedure was performed. Based on an iterative Markov chain Monte Carlo (MCMC) method 20 data sets were generated to provide estimates. MAR or MNAR was assumed (50). Statistical significance was defined as *p* < 0.05 (2-tailed) for all analysis. All analyses were performed with the Statistical Package for Social Science SPSS Version 25 (51).

The standard method to calculate scale scores for the INSPIRE based on the INSPIRE Scoring instructions (52) were applied. Thus, items were recoded from 0 to 4 and per subscale the average support was calculated. These scores were, then multiplied by 25 to create a scale ranging from 0 to 100. The scale scores for the WHODAS 2.0 were calculated based on the scoring instructions provided by the WHO (47) but without the restriction on missing values for each domain, meaning everyone with valid answer was included.

Multilevel regression models were used to test the relation between the WHODAS 2.0 (dependent variable) and INSPIRE adjusted for service user characteristics (age, gender, marital status, highest education, and income) and health status (main diagnosis, mental health history), with a random intercept for the sites as the hierarchically higher level.

## Results

### Description of the Study Population

Across the five sites, in total 931 participants were included, female and male almost equally represented (51.9 and 47.9%, respectively). Participants were on average 46.5 (SD = 12.6) years old. The majority were single or married/with partner (44.8 and 33.9%, respectively). Almost a third of the participants stated to be retired (29.1%), nearly a quarter reported to be not employed but looking for work, only a few were employed full-time (17.4%), and some stated to have side jobs (3.4%). The majority of participants had a mental health service use history of more than 4 years (67.0%). Almost half of the participants reported high school or equivalent as highest education status (43.7%). Around a third of the participants reported to have either no income (36.4%) or an income below average (33.4%). The majority of the study population was diagnosed with schizophrenia (60.8%) (Supplementary Material).

### Perceived Support for Recovery

The average Support Score across the sites was 72.5%, indicating that the support provided by the mental health care workers was just at the line of being supportive toward recovery. The Relationship Score of the INSPIRE across all sites demonstrated that the service users valued the relationship with their mental health workers (80.3%) (Table 1). The sub-scale rating of the Support Score shows which category of the recovery process can be improved if support is not perceived well. The Connectedness sub-scale shows how important it is for mental health service users to be supported by other people, to have positive relationships, to be supported by other service users, and to be a part of the community.

**Table 1:** INSPIRE scores per project site and for the total sample.

**INSPIRE Mean (SD)**	**Zagreb, Croatia**	**Kotor, Montenegro**	**Suceava, Romania**	**Skopje, North Macedonia**	**Sofia, Bulgaria**	**Total**
**Project sites**						
**INSPIRE, sub-scores; Mean (SD)**						
Support score total^*^	88.96 (15.43)	71.31 (16.98)	75.83 (12.73)	63.64 (15.80)	64.49 (25.23)	72.5 (20.1)
Relationship score total^*^	90.76 (15.26)	82.73 (14.02)	80.37 (10.47)	76.82 (15.79)	71.91 (20.89)	80.3 (16.9)
**Support score sub-scale rating; Mean (SD)**						
**Connectedness**	86.26 (19.41)	71.17 (19.74)	73.29 (12.69)	62.79 (20.11)	64.45 (26.77)	71.3 (21.9)
**Hope**	92.49 (14.01)	74.87 (20.43)	74.94 (15.47)	65.12 (20.82)	66.91 (27.07)	74.6 (22.4)
**Identity**	86.48 (19.03)	68.58 (20.32)	77.29 (14.31)	62.43 (21.51)	64.39 (26.51)	71.5 (22.6)
**Meaning and purpose**	90.86 (16.98)	72.67 (19.63)	78.23 (14.69)	66.41 (16.84)	64.88 (26.38)	74.2 (21.6)
**Empowerment**	89.09 (17.82)	70.20 (18.34)	75.35 (15.67)	62.00 (17.58)	61.81 (28.19)	71.4 (22.5)

* *Support sub-scale, scores over 72% indicate that support provided is perceived as helpful; Relationship sub-scale, scores over 78% indicate that the relationship is perceived as valuable to the service user; reported are mean and standard deviation*.

### Country-Specific Perceived Support for Recovery

The sub-scale rating for Connectedness showed that service users in North Macedonia and Bulgaria do not feel well-supported (62.79 and 64.45%, respectively). Hope refers to feeling hopeful about the future, believing that one can recover, feeling motivated to make changes, and having hopes and dreams. The results of the Hope sub-scale rating showed that there is room for improvement in North Macedonia (65.12%) and Bulgaria (66.91%). The sub-scale rating for Identity for Montenegro (68.58%), North Macedonia (62.43%), and Bulgaria (64.39%) indicated that it is an important part of recovery for the service user to be able to deal with stigma, to feel good about themselves. The sub-scale Meaning and Purpose showed that service users in North Macedonia and Bulgaria indicated that they would like to receive more support regarding the understanding of their mental health illness, things they like to do, rebuilding their life after difficult experiences, and quality of life (66.41 and 64.88%, respectively).The sub-scale Empowerment represents how service users can be supported to feel empowered, e.g., having their life under control, being able to manage their mental illness, trying new things, and to build on their strengths. The results for the sub-scale Empowerment showed that service users in Montenegro (70.20%), in Macedonia (62.00%), and in Bulgaria (61.81%) indicated that they would like to be more supported in this area (Table 1).

### Level of Functioning (WHO-DAS)

The distribution of WHODAS 2.0 scores showed some variation across sites. The highest degree of functional limitations for the total sample was in the domain participation (mean score 43.8), the lowest degree of functional limitation was in the domain self-care-hygiene (mean score 15.8) (Table 2).

**Table 2:** WHO Disability Assessment Schedule 2.0 sub-scales per project site and for the total sample.

**WHODAS 2.0**	**Zagreb**,	**Kotor**,	**Suceava**,	**Skopje**,	**Sofía**,	**Total**
sub-scales^*^	**Croatia**	**Montenegro**	**Romania**	**North Macedonia**	**Bulgaria**	
**Mean (SD)**						
**Cognition**	38.2 (27.7)	19.3 (23.8)	30.7 (18.8)	27.9 (21.9)	42.1 (23.1)	31.6 (24.5)
**Mobility**	21.6 (25.3)	17.6 (25.5)	20.1 (19.9)	25.8 (24.9)	23.7 (24.0)	21.7 (24.1)
**Self-care-hygiene**	15.7 (21.7)	9.3 (16.4)	15.9 (18.3)	17.5 (22.2)	20.9 (23.0)	15.8 (20.8)
**Getting along**	38.8 (27.6)	15.8 (18.0)	38.5 (20.9)	30.1 (24.8)	39.5 (19.8)	32.3 (24.1)
**Life activities (Household)**	41.4 (33.1)	22.1 (25.7)	31.2 (22.5)	33.6 (26.9)	28.9 (25.6)	33.3 (27.6)
**Life activities (School/Work)**	61.7 (37.2)	18.9 (24.4)	27.6 (20.0)	31.1 (27.1)	40.0 (27.1)	35.6 (29.2)
**Participation**	48.2 (28.2)	34.8 (19.9)	49.0 (17.8)	41.6 (21.6)	46.0 (18.9)	43.8 (21.6)
**Total WHODAS 2.0**	36.4 (20.6)	21.5 (17.2)	32.8 (16.2)	30.9 (19.7)	37.1 (17.4)	31.6 (19.1)

* *0, no disability; 100, full disability*.

### Associations Between INSPIRE and WHODAS 2.0

Adjusted for individual characteristics, the support received by a mental healthcare provider (*b* = −0.10, *p* = 0.011) and the relationship between service user and service provider (*b* = −0.13, *p* = 0.003) were both related to the degree of functioning. We found that for every 10 points more on the support or relationship scale, functional limitations decreased with 1 and 1.3 points, respectively. These results imply that service users who perceive the support as helpful and who value the relationships with healthcare providers tend to have less functional limitations (Table 3).

**Table 3:** Multilevel regression model with WHODAS 2.0 as dependent variable.

**Dependent WHODAS 2.0 Predictor**		**95% CI**		*p*-value^*^
	**Estimate**	**Lower**	**Upper**	
		**bound**	**bound**
Age	0.08	−0.04	0.20	0.192
Sex: Female	4.45	1.94	6.96	0.001
**Main diagnosis**				
Diagnosis: Bipolar disorder	2.13	−1.67	5.93	0.273
Diagnosis: Severe Depression	3.94	0.66	7.22	0.019
Reference: Schizophrenia				
**Mental health history**				
Mental Health History <2 years	−6.18	−9.67	−2.69	0.001
Mental Health History 2–4 years	−3.38	−6.91	0.15	0.061
Reference: More than 4 years				
**Marital status**				
Single	2.08	−1.10	5.26	0.200
Widowed	1.17	−4.90	7.24	0.706
Separated or Divorced	4.36	0.42	8.29	0.030
Reference: Married				
**Highest education**				
High School	−2.84	−6.19	0.52	0.098
Vocational Training	−2.69	−6.97	1.59	0.218
College and higher	−5.96	−9.92	−2.00	0.003
Reference: Grammar School				
**Income**				
Not employed	3.14	0.05	6.23	0.047
Income average	−3.68	−7.45	0.10	0.056
Income above average	−3.19	−9.50	3.12	0.322
Reference: below average				
**INSPIRE**				
INSPIRE_Support subscale 100	−0.10	−0.18	−0.02	0.011
INSPIRE_Relationship subscale 100	−0.13	−0.22	−0.05	0.003

* *Statistical significance was defined as p < 0.05*.

Regarding individual characteristics, the following associations were found. Participants who identified as female indicated a lower overall level of functioning compared to those identifying as male (*b* = 4.45, *p* = 0.001). Participants with severe depression evaluated their degree of functional limitations lower compared to participants with schizophrenia (*b* = 3.94, *p* = 0.019). The shorter the history of mental health service use, the lower the degree of functional limitations (*b* = −6.18, *p* = 0.001). Being divorced or separated was also associated with a lower degree of functioning (*b* = −4.36, *p* = 0.030). Participants with a college degree or higher evaluated their degree of functional limitations lower compared to those who reported their highest educational degree to be grammar school (*b* = −5.96, *p* = 0.003). Level of income was also associated with the degree of functioning. Participants who stated that they were not employed, indicated a higher degree of functional limitations compared to those with an income below average (*b* = 3.14, *p* = 0.047).

## Discussion

The aim of this study was to analyse the level of functioning and the perceived support for the recovery process among a large population of individuals with SMI across five mental health centers in five countries in Central and Eastern Europe, and to explore associations between perceived support for recovery and the degree of functional limitations. The study showed that across all mental health centers, the greatest functional limitations were in the domain *participation in society*, followed by life activities such as managing one's own household or going to school or pursue a career. Most service users were satisfied with the recovery support they received from their mental health care provider and that they valued the relationship with providers, but some service users perceived support as less enabling for recovery. Moreover, service users who evaluated the support they receive by their healthcare provider as valuable and who reported to have a meaningful relationship with them had a lower degree of functional limitation.

The degree of functional limitations (measured with WHODAS 2.0) in the sample was comparable with other study populations with severe mental illness (44, 53). In this study, participants indicated the highest degree of functional limitation in the domains *participation in society, work*, and *household*, followed by *getting along with people*. These results are consistent a prior study with over 65,000 respondents, where people with mental illnesses showed the highest degree of functional limitations in the domains *household, participation in society*, and *work*, followed by *getting along with people, understanding and communicating, getting around*, and *self-care* (44). Of note, participants with severe depression indicated higher levels of functional limitations than service users with schizophrenia. These results are inconsistent with those by Kohn et al. (9), which found that people with schizophrenia experienced the highest levels of functional limitations. However, this study was carried out in India with a different mental health system infrastructure and used different measures of functional limitation. We also found that a higher educational status and paid employment were associated with higher level of functioning. There are numerous studies showing the positive effects of employment on mental health outcomes (54); however, a recent systematic review also found that employment was associated with a higher level of functioning (49). Given the role that these areas of life play in the personal and social recovery, it remains relevant to continue to examine the effects of people with obtaining meaningful employment or starting education on functioning, particularly over time (23).

Research shows that the earlier one accesses treatment, the better the odds of recovery/remission and the greatest odds of having a less severe form of the illness and/or symptoms (55–57). The sample largely comprised of service users who were in the mental health care system for several years. We did not evaluate if the onset of the mental illness is associated with the history of use service and therefore with the degree of functional limitation. Prior research has found that a longer length of stay in a psychiatric hospital does adversely impact social integration (7), particularly employment and housing outcomes.

Our study showed that service users across sites were generally satisfied with the support received from their mental health care providers. A recent study from Norway which also used the INSPIRE tool found that service users who experienced higher perceived support for personal recovery from their health care provider are more satisfied with the care they receive (53). Nevertheless, there is room for improvement across all centers of the project. Further embedding and sustaining recovery-oriented principles and care approaches in clinical care is an important point of attention to consider in RECOVER-E countries, many of which are undergoing mental health system transformations. Thus, the findings of this study may help to shape their way of providing recovery-oriented care and improve how mental health service users evaluate the support they receive.

Research shows that social support and a social network is of importance for people with mental health problems (58). An adequate social network may reduce the need for mental health services or pave the way to access to professional help (58). The findings of this study showed that being in a relationship was associated with a greater perception of recovery support. It may be that those with personal support and/or a social network may generally be more positive toward support from their mental health providers as well or the importance of a personal network or personal relationship in benefitting from a supportive professional relationship. Previous studies have shown that the severity of the mental illness was related to the size of the social network; greater severity of symptoms was associated with a smaller social network (59).

The relationship subscale scores of the INSPIRE were evaluated to a higher degree than the support subscale across all sites. This seems to suggest that service users perceived the overall relationship with their mental health providers as positive but perceived the actual support they received less favorably. The support mental health service provider provide could be improved by offering interventions aimed at the three areas of recovery: symptomatic, personal and recovery within the service user's social context. Connectedness, identity, and empowerment subscales were scored lowest among the support scale among all sites. This points to the importance of ensuring that service delivery models incorporate components of recovery of identify and opportunities for personal empowerment and reflect preferences and goals on these two domains in recovery plans.

### Strengths and Limitations

The participating countries in this study are traditionally underrepresented in academic research, particularly in research on real-world health care. A clear strength is the large study population of people with severe and enduring mental health problems, who may be difficult to engage in research. Nevertheless, some limitations need to be acknowledged. The study is based on the baseline measurement in a clinical trial and thus essentially cross-sectional, which means that causality underlying associations remains uncertain. The data comprised of self-reports, thus they might be biased by distorted perceptions and social pressure. Although inclusion criteria were the same in all sites, due to different settings, they may have been applied variably across sites, resulting in different study populations. Therefore, descriptive statistics and comparisons between countries should be interpreted with caution. Estimations of the size of associations are probably less affected, but the confidence intervals of estimations may be affected by the representation of subgroups.

### Future Research and Implications

This paper provides insight into the degree of functional limitations and confirms that it is associated with perceived support for recovery. Future studies should explore the underlying causal mechanisms, using longitudinal study designs. In the RECOVER-E project, we have implemented and will evaluate the cost-effectiveness of a community-based team for recovery-orientated treatment of people with severe mental illness. This will enable to examine the pathway that starts with recovery-orientated treatment, which results in user perceptions of recovery support, and finally leads to fewer functional limitations. In addition, we will explore the outcomes in subgroups of users to determine whether specific subgroups receive or benefit more from recovery support, including subgroups defined by initial degree of functional limitations. The latter helps help to examine the reversed pathway: starting with a status with few functional limitations, people may be able to receive or perceive more recovery support.

This baseline data has been and will continue to be used to inform mental health delivery in each of the study sites; results are used to improve care delivery to service users, document service user needs for care, and shape how community mental health providers work together as a team and as individuals to provide care. Further, the results have been shared with policymakers and other stakeholders through local publications, conferences, and strategy meetings in order to improve infrastructures for care, mental health guidelines, and mental health policies in each of the countries involved in the study. This dissemination of results to improve the knowledge to practice gap will continue as more data is collected and more is understood about the impact of the RECOVER-E intervention on patient outcomes, provider outcomes, cost effectiveness, and of the implementation of the intervention in each site.

## Conclusion

This study is one of the first studies to investigate perceived quality of recovery-oriented support among service users with severe mental illness in Central and Eastern Europe and its association with functional limitation in five different countries. We found that, the perceived support that service users receive from their healthcare providers and the relationship between professional and service user has an impact on the degree of functional limitations. Therefore, increasing the availability of the mental health services and the relationship with the users, by the introduction of the CMHT models, which incorporate these principles, may improve the functional outcome of the service user with severe mental health illness.

## Trial Registration

Each trial was registered before participant enrolment in the clinicaltrials.gov database: Croatia, Zagreb (Trial Reg. No. NCT03862209); Montenegro, Kotor (Trial Reg. No. NCT03837340); Romania, Suceava (Trial Reg. No. NCT03884933); Macedonia, Skopje (Trial Reg. No. NCT03892473); Bulgaria, Sofia (Trial Reg. No. NCT03922425).

## Data Availability Statement

The datasets presented in this article are not readily available because data is protected by the European Data Protection Law but maybe available by the corresponding author on reasonable request. Requests to access the datasets should be directed to Michel Wensing, (michel.wensing@med.uni-heidelberg.de).

## Ethics Statement

The studies involving human participants were reviewed and approved by Medical Ethics Committee of the Medical Faculty Heidelberg (S-496/2018). The patients/participants provided their written informed consent to participate in this study.

## Author Contributions

IP and LS-Z conceived this project. MW and LS-Z conceived this study and elaborated the research protocol. CR and JK analyzed the data and wrote the first draft of the manuscript. All authors provided substantial comments and approved the final version of the manuscript.

## Funding

This project has received funding from the European Union's Horizon 2020 research and innovation programme under Grant Agreement 779362.

## Conflict of Interest

The authors declare that the research was conducted in the absence of any commercial or financial relationships that could be construed as a potential conflict of interest.

## Publisher's Note

All claims expressed in this article are solely those of the authors and do not necessarily represent those of their affiliated organizations, or those of the publisher, the editors and the reviewers. Any product that may be evaluated in this article, or claim that may be made by its manufacturer, is not guaranteed or endorsed by the publisher.

## Abbreviations

WHO: World Health Organization
CMHT: Community Mental Health Team
SMI: Severe Mental Illness
RECOVER-E: Large-scale implementation of community-based mental health care for people with severe and enduring mental ill health in Europe
WHODAS 2.0: World Health Organization Disability Assessment Schedule 2.0.
