# Supplementary material for: Perceived Support for Recovery and Level of Functioning Among People With Severe Mental Illness in Central and Eastern Europe: An Observational Study
Source: Front Psychiatry. 2021 Sep 21;12:732111. doi: 10.3389/fpsyt.2021.732111 (PMC8490702; doi:10.3389/fpsyt.2021.732111)
Supplement: Supplementary file 1 [file Data_Sheet_1.PDF]

Supplementary Material

Supplementary Material 1: Description of the study population per study site

	Zagreb, Croatia N=169 (18.2%)	Kotor, Montenegro N=202 (21.7%)	Suceava, Romania N=180 (19.3%)	Skopje, North Macedonia N=180 (19.3%)	Sofia, Bulgaria N=200 (21.5%)	Total study population N=931 (100%)
**Sex n, %**						
Female	88 (52.1)	89 (44.1)	99 (55.0)	106 (58.9)	101 (50.5)	483 (51.9)
Male	80 (47.3)	113 (55.9)	81 (45.0)	74 (41.1)	98 (49.0)	446 (47.9)
Other	1 (0.6)	0	0	0	1 (0.5)	2 (0.2)
**Age (Mean, SD)**						
	41.2 (12.6)	48.7 (12.2)	52.6 (12.2)	46.1 (11.7)	43.9 (11.6)	46.5 (12.6)
**Marital Status n, %**						
Single	111 (65.7)	90 (44.6)	44 (24.4)	69 (38.3)	103 (51.3)	417 (44.8)
Widowed	9 (5.3)	8 (4.0)	13 (7.2)	5 (2.8)	6 (3.0)	41 (4.4)
Separated	8 (4.7)	4 (2.0)	2 (1.1)	2 (1.1)	10 (5.0)	26 (2.8)
Divorced	6 (3.6)	30 (14.9)	15 (8.3)	17 (9.4)	17 (8.5)	85 (9.1)
Married/with partner	34 (20.1)	68 (33.7)	98 (54.4)	84 (46.7)	32 (16.0)	316 (33.9)
Prefer not to say	1 (0.6)	1 (0.5)	8 (4.4)	3 (1.7)	32 (16.0)	45 (4.8)
**Employment Status n, %**						
Full-time	41 (24.3)	26 (12.9)	14 (7.8)	62 (34.4)	19 (9.5)	162 (17.4)
Part-time	1 (0.6)	11 (5.4)	3 (1.7)	9 (5.0)	8 (4.0)	32 (3.4)
Side job	1 (0.6)	1 (0.5)	0	4 (2.2)	1 (0.5)	7 (0.8)
Not employed, looking for work	39 (23.1)	54 (26.7)	4 (2.2)	40 (22.2)	59 (29.5)	196 (21.1)
Not employed, not looking for work	37 (21.9)	29 (14.4)	16 (8.9)	34 (18.9)	12 (6.0)	128 (13.7)
Retired	28 (16.6)	57 (28.2)	131 (72.8)	24 (13.3)	31 (15.5)	271 (29.1)
Disabled	8 (4.7)	19 (9.4)	0	0	24 (12.0)	51 (5.5)
Student	11 (6.5)	2 (1.0)	0	3 (1.7)	7 (3.5)	23 (2.5)
Prefer not to say	0	0	10 (6.6)	1	33 (16.5)	44 (4.7)
Other	3 (1.8)	2 (1.0)	2 (1.1)	3 (1.7)	6 (3.0)	16 (1.7)
**Mental Healthcare History n, %**						
less than one year	12 (7.1)	13 (6.4)	16 (8.9)	25 (13.9)	18 (9.0)	84 (9.0)
between one and two years	3 (1.8)	14 (6.9)	11 (6.1)	18 (10.0)	13 (6.5)	59 (6.3)
between two and three years	12 (7.1)	13 (6.4)	19 (10.6)	18 (10.0)	9 (4.5)	71 (7.6)
between three and four years	14 (8.3)	12 (5.9)	12 (6.7)	8 (4.4)	12 (6.0)	58 (6.2)
more than four years	127 (75.1)	147 (72.8)	122 (67.8)	111 (61.7)	117 (58.5)	624 (67.0)
Prefer not to say	0	1 (0.5)	0	0	31 (15.5)	32 (3.2)

	Croatia<br>169 (18.2)	Montenegro<br>202 (21.7)	Romania<br>180 (19.3)	North Macedonia<br>180 (19.3)	Bulgaria<br>200 (21.5)	Total<br>931 (100)
**Highest Education status n, %**						
Grammar School	19 (11.2)	39 (19.3)	73 (40.6)	17 (9.4)	25 (12.5)	173 (18.6)
High school or equivalent	79 (46.7)	107 (53.0)	50 (27.8)	99 (55.0)	72 (36.0)	407 (43.7)
Vocational Training	21 (12.4)	29 (14.4)	33 (18.3)	10 (5.6)	21 (10.5)	114 (12.2)
Some College	6 (3.6)	1 (0.5)	2 (1.1)	1 (0.6)	5 (2.5)	15 (1.6)
College graduate (4 years)	16 (9.5)	10 (5.0)	6 (3.3)	0	1 (0.5)	33 (3.5)
Associate degree	6 (3.6)	2 (1.0)	1 (0.6)	3 (1.7)	0	12 (1.3)
Bachelor degree	7 (4.1)	5 (2.5)	0	39 (21.7)	16 (8.0)	67 (7.2)
Master's degree	8 (4.7)	3 (1.5)	4 (2.2)	5 (2.8)	21 (10.5)	41 (4.4)
Professional degree	6 (3.6)	2 (1.0)	11 (6.1)	3 (1.7)	3 (1.5)	25 (2.7)
Doctorate/PhD	1 (0.6)	0	0	0	0	1 (0.1)
Other	0	0	0	3 (1.7)	4 (2.0)	7 (0.8)
Prefer not to say	0	1 (0.5)	0	0	32 (16.0)	33 (3.5)
**Income n, %**						
No income	95 (56.2)	74 (36.6)	16 (8.9)	71 (39.4)	83 (41.5)	339 (36.4)
Below average	35 (20.7)	82 (40.6)	109 (60.6)	45 (25.0)	41 (20.5)	312 (33.4)
Average	33 (19.5)	34 (16.8)	6 (3.3)	42 (23.3)	29 (14.5)	144 (15.5)
Above average	3 (1.8)	9 (4.5)	1 (0.6)	16 (8.9)	7 (3.5)	36 (3.9)
Prefer not to say	3 (1.8)	1 (0.5)	48 (26.7)	5 (2.8)	40 (20.0)	97 (10.8)
**Primary diagnosis n, %**						
Schizophrenia (F20 – F29)	124 (73.4)	133 (65.8)	80 (44.4)	75 (41.7)	154 (77.0)	566 (60.8)
Bipolar disorder (F30,F31)	11 (6.5)	19 (9.4)	15 (8.3)	35 (19.4)	34 (17.0)	114 (12.2)
Severe Depression (F32,F33)	23 (13.6)	50 (24.8)	73 (40.6)	70 (38.9)	12 (6.0)	228 (24.5)
Other	0	0	7 (3.9)	0	0	7 (0.8)
No answer	11 (6.5)	0	5 (2.8)	0	0	16 (1.7)
