# Supplementary material for: Perceived Support for Recovery and Level of Functioning Among People With Severe Mental Illness in Central and Eastern Europe: An Observational Study
Source: Front Psychiatry. 2021 Sep 21;12:732111. doi: 10.3389/fpsyt.2021.732111 (PMC8490702; doi:10.3389/fpsyt.2021.732111)
Supplement: Supplementary file 2 [file Data_Sheet_2.docx]

**Supplementary material 2.**

| **Site** | **Description of care system** |
| --- | --- |
| Zagreb, Croatia | Specialised mental health care is provided primarily within hospitals (both in inpatient and outpatient settings). Pilot community mental health teams are being tested in several parts of Croatia, although not at this particular site. There is a community mental health centre in the city of Zagreb, which is broader in scope than the multidisciplinary community mental health team's work and focuses primarily on common mental disorders such as depression and anxiety. The primary interventions delivered as part of usual care include psychotherapy and medication. Treatment options available are dependent on the training and education of the provider. |
| Kotor, Montenegro | For people with severe mental illness, inpatient care is provided at this implementation site by the Psychiatric Hospital in Kotor, an outpatient care is provided by the mental health centre in Kotor (which is based in the primary care structure of the health system). The mental health centre accepts patients in its outpatient clinic and does not provide home-based treatment or crisis resolution outside of the clinic. The primary intervention that the mental health centre provides is pharmacological intervention. Psychosocial rehabilitation is provided occasionally but not systematically. Inpatient admission is the standard procedure for any deterioration in mental health status and more than half of the beds in the psychiatric hospital are occupied by long-term care clients with severe mental illness from across the whole country. |
| Suceava County, Romania | Inpatient admission is the standard protocol during any deterioration in mental health status for people with severe mental illness. There are currently no community mental health teams to provide home-based treatment or services in the community. The hospital has an occupational therapist but they do not routinely work with people with SMI. |
| Skopje, North Macedonia | For people with severe mental illness, inpatient care is provided by the University Clinic of Psychiatry in Skopje (the nation's capital city) by the psychiatric hospital in Skopje or in a psychiatric ward in the city's general hospital. Specialised outpatient care is provided by a network of outpatient clinics. There are a few community mental health centres in North Macedonia. The community mental health centres accept patients in the office on an outpatient basis and do not provide home-based treatment or crisis resolution outside of the clinic. The primary intervention that the team provides is dispensing medication, supportive psychotherapy, psychosocial support and psychoeducation. Inpatient admission is the standard protocol during any deterioration in mental health status for clients with SMI. |
| Sofia, Bulgaria | People with SMI primarily receive medication from a psychiatrist in an outpatient department of the hospital. There are limited evidence-based psychosocial interventions provided to people with SMI and inpatient admission is the standard protocol during any deterioration in mental health status for clients with SMI. |
| Note. This information is identical to Table 2 in the published study protocol (34). | |
